# Human Health Risk Assessment of Heavy Metals and Metalloids in Herbal Medicines Used to Treat Anxiety: Monitoring of Safety

**DOI:** 10.3389/fphar.2021.772928

**Published:** 2021-11-10

**Authors:** Ana C. R. Geronimo, Elaine S. P. Melo, Kassia R. N. Silva, Hugo S. Pereira, Valdir A. Nascimento, David J. Machate, Valter A. do Nascimento

**Affiliations:** ^1^ Group of Spectroscopy and Bioinformatics Applied Biodiversity and Health (GEBABS), School of Medicine, Federal University of Mato Grosso do Sul, Campo Grande/MS, Campo Grande, Brazil; ^2^ Federal University of Mato Grosso do Sul, Campo Grande/MS, Campo Grande, Brazil

**Keywords:** herbal medicines, toxicity, heavy metals, anxiety, metalloids

## Abstract

The present study estimated the human health risk assessment and daily intake of heavy metals and metalloids in herbal medicines used to treat anxiety in Brazil. Six different brands of herbal medicines were purchased in the city of Campo Grande/MS, Brazil: Pasalix^®^, Calman^®^, Serenus^®^, Maracugina^®^, Prakalmar^®^ and Calmasyn^®^. In total, eight elements including As, Ba, Cd, Co, Cr, Cu, Fe, and Pb were analyzed using optical emission spectrometry with inductively coupled plasma (ICP OES). Only the concentration of As in the herbal medicine Prakalmar^®^ is above the values established by United States Pharmacopoeia Convention (USP) and Brazilian Pharmacopoeia (BF) for permitted concentration of elemental impurities in drugs substances. The concentration of Ba, Cd, Co, Cr and Cu in all herbal medicines are lower than the values set by USP and FB. The concentration of Pb in Calman^®^ is lower than the limits established by BF, but higher than those established by USP. Pasalix, Serenus^®^, Calmasyn^®^, Prakalmar^®^ and Marcacugina^®^ have a higher Pb concentration than the values allowed by USP and BF. All herbal medications have concentrations within safe ranges for human consumption, with the exception of Calmasyn^®^, which has Pb over the value defined by USP for oral permissible daily exposure (PDE) for elemental impurities. The values of estimated daily intake (*EDI*) of metal (loid)s in adults and children obtained from the consumption of the herbal medicines are below the values stipulated by the minimal risk levels (MRLs). All the hazard index (*HI*) values recorded in this study were below 1. However, monitoring by regulatory agency is necessary, large doses of heavy metal may cause acute or chronic toxicities.

## Introduction

Depression and other Common Mental Disorders are global health problems (World Health Organization, 2017). In this regard, the anxiety disorders (generalized anxiety disorder, panic disorder/agoraphobia, social anxiety disorder, and others) are burdensome to communities. Benzodiazepines (BZs) are sedatives used to treat many kinds of anxiety disorders, specifically panic disorder with or without agoraphobia, generalized anxiety disorder, and social anxiety disorder. However, possible abuse, abstinence and dependence can occur during the use of this medication (Balon et al., 2020).

Due to the effects caused by some medications, high prices, people seek to use medicinal plants or herbal remedies to treat various types of diseases including anxiety. In fact, according to a monograph published by European Medicines Agency on *Passiflora incarnata*, several countries as Austria, Belgium, France, Germany, Spain, Sweden and United Kingdom use this plant for temporary relief of mild anxiety and to aid sleep (European Medicines Agency, 2014). Currently there are a large number of herbal medicines used in the treatment of anxiety based on plants such as Passiflora Incarnata and Passiflora together with Crataegus and Salix alba (compound) (Nascimento et al., 2009), which are commercialized in several pharmacies of Brazil as Calman^®^, Pasalix^®^, Serenus^®^, Calmasyn^®^, Prakalmar^®^ and Maracugina^®^.

To date, no reports of clinical studies have been found on the toxicity of herbal medicines based on *Passiflora* in Brazil or in other countries. In fact, clinical studies have shown that Pasalix^®^ does not cause toxicity (Nascimento et al., 2009), however, studies on heavy metals, metals or metalloids in their composition and in Calman^®^, Serenus^®^, Calmasyn^®^, Prakalmar^®^ and Maracugina^®^ are scarce. Some species of medicinal plants accumulate metal (loid)s in their composition that can cause health damage (Street, 2012; Rocha et al., 2019). The anxiety and depression may be related to a plausible high exposure to heavy metals in the contaminated areas, which are specifically industrialized zones (Jaga and Dharmani, 2007; Ayuso-Álvarez et al., 2019). In addition, there is a relationship between exposure to heavy metals and anxiety (Berk et al., 2014; Theorell et al., 2015). Clinical studies showed that patients with depression and panic attacks disorders showed an excess of some metals concentration such as cadmium (Cd) and lead (Pb) (Jurczak et al., 2018). In the reserpine induced mouse model of depression, the subchronic arsenic (As) exposure induces the anxiety-like behaviors in normal mice and enhances the depression-like behaviours (Chang et al., 2015). Cobalt (Co) intoxication may cause panic-anxiety attacks, coughing, and difficulty in breathing, interstitial lung disease, etc (Barceloux and Barceloux, 1999). According studies, there is decreased Zinc (Zn) and increased Copper (Cu) in individuals with anxiety (Russo, 2011). Review studies present important results from preclinical and clinical studies showing involvement of essential elements such as iron (Fe) and chromium (Cr) in depression and anxiety (Młyniec et al., 2014). On the other hand, Cr supplementation may relieve symptoms in people with a mood disorder known as atypical depression (Docherty et al., 2005).

Chemical elements are not simply related to anxiety and depression, they can also be toxic to humans. Among the several dangerous toxics, Cd induces various epigenetic changes in mammalian cells *in vivo* and *in vitro*, causing the development of various types of cancers (Godt et al., 2006). According to World Health Organization (WHO), Pb is harmful to adults and young children and causes anemia, hypertension, renal impairment, immunotoxicity and toxicity to the reproductive organs [World Health Organization (WHO), 2021]. Based on the available evidence, the long-term exposure to arsenic due to ingestion of food can cause cancers of the skin, lungs, and bladder (Hong et al., 2014). Barium (Ba) is not considered to be an essential element for human nutrition. However, the toxicity of individual barium salts depends on their solubility. The Ba^2+^ ion and the soluble compounds of barium are toxic to humans. In poisoning, it blocks passive efflux potassium channels without affecting the Na/K-ATPase pump, which may lead to significant hypokalemia (Tao et al., 2016). Another chemical element that is toxic to humans is Cr. Since Cr(III) is poorly absorbed by any route, Cr(VI) is much more toxic than the trivalent form, it can be absorbed by the skin, lung and gastrointestinal tract (Sun et al., 2015). In fact, species as Cr(VI), Cr(V), Cr(IV) and ultimately, Cr(III) could attack DNA, proteins, and membrane lipids, thereby disrupting cellular integrity and functions (De Mattia et al., 2004). Cr(VI) is considered a human carcinogen and toxic to many plants (International Agency for Research on Cancer, 1990; Shanker et al., 2005). Unlike some chemical elements, iron is an essential element for almost all living organisms. However, iron toxicity from intentional or accidental ingestion is a common poisoning among children. According to the study, high iron intake can result in severe toxicity (Yuen and Becker, 2021). Chronic or long-term exposure to high quantities of copper via contaminated food and water sources can cause copper poisoning. Diarrhea, hemolytic anaemia, and migraines are all symptoms of this disorder, and in severe cases, acute renal failure can develop, resulting in permanent kidney damage (National Research Council (US) Committee on Copper in Drinking Water, 2000). Cobalt is a naturally occurring element found in water, plants and animals. Cobalt has a biologically necessary role as metal constituent of vitamin B12, however, excessive exposure has been shown to induce various adverse health effects as neurological, cardiovascular and endocrine deficits (Leyssens et al., 2017).

In addition to all these above-mentioned factors, herbal medicines are popular due to their easy access to purchase without a prescription. Thus, several factors have become a challenge associated with the effective monitoring of the safety of herbal medicines. According to World Health Organization (WHO), “the safety of traditional and herbal medicines has therefore become a major concern for both national health authorities and the general public” (WHO, 2004).

To guarantee the safety of consumers due to the ingestion of pharmaceutical products, regulations for elementary impurities have been established in some countries e.g., the United States via USP (USP, 2021). The most toxic and ubiquitous class 1 elements (Cd, As and Pb), as well as class 2 (Co) and class 3 (Ba, Cr, Cu) must be considered in the risk assessment for all drug products (USP, 2021). The regulatory requirements for the registration of herbal medicines products in Brazil are similar to those in Europe (da Fonseca et al., 2020), however, the limit for heavy metals, metals and metalloids in medicinal plants *in natura* not yet established.

Clinical data and research involving toxicity in humans due to ingestion of heavy metals and metalloids in herbal medicines are scarce. However, there are studies that consider health risk assessment, such as chronic daily intake (*CDI*) and non-cancerous risks due to the intake of heavy metals (metalloids) assessed by the target risk quotient (*THQ*) and hazard index (*HI*) (Brima, 2017; de Souza et al., 2021). Thus, in order to obtain a health risk assessment, calculations must be performed considering the *CDI* and *THQ* (Brima, 2017; de Souza et al., 2021). Risk assessment is an evaluative and comparative process. Thus, risk evaluation involves comparing the results of the risk analysis to specified risk criteria decide where extra action is needed (US Environmental Protection Agency, 2021).

To date, no research has been conducted on chronic daily intake (*CDI*) and the target risk quotient (*THQ*) of herbal medicines investigated in this article. Although the *Passiflora incarnata* L. species is used in the food, cosmetic and pharmaceutical industries and included in many Pharmacopoeias (da Fonseca et al., 2020), it can contain toxic chemical elements in their composition. Therefore, there is a need to monitor the levels of heavy metals, metals and metalloids in herbal medicines consumed and marketed in Brazil and other countries.

This study aims to investigate the levels of potentially toxic elements Co (Cobalt), Cu (Copper), Cr (Chrome), Fe (Iron), As (Arsenic), Ba (Barium), Cd (Cadmium) and Pb (Lead) and also calculate the CDI and total hazard index (*HI*) in herbal medicines commonly consumed in Brazil to assess their health risk. In fact, the metal concentrations in herbal medicines were compared with established permissible limits. Also a daily intake of herbal medicines by human were calculated and compared with the recommended dietary intakes. The estimated daily intake (*EDI*) values of elements in adults and children (µg/kg/day) through the consumption of herbal medicines were compared with minimal risk levels (MRLs). Finally, the hazard quotients for each studied metal and a hazard index was used to quantify the non-cancer risk.

## Materials and Methods

### Reagents and Standard Solution

All the chemicals and reagents used were of analytical grade. All laboratory material was decontaminated in a 10% HNO3 solution bath (v v-1) for 24 h and rinsed with ultrapure water and subsequently dried in a clean environment at room temperature. Nitric acid 65%, HNO_3_ (Merck - Darmstadt, Germany), hydrogen peroxide 30%, H_2_O_2_ (Merck - Darmstadt, Germany) and ultrapure water (18 MΩcm, Milli-Q Millipore, Bedford, United States) were used for the digestion of the samples. Standard solutions (Specsol, Brazil, 1,000 mg/L in 2% HNO_3_) of As, Ba, Cd, Co, Cr, Cu, Fe and Pb were used to prepare the calibration curves.

### Sample Collection

The 180 samples of herbal medicine commercially available in the form of pills were purchased from local drugsores in the city of Campo Grande/MS, Brazil. Six different batches of six manufacturers of herbal medicines were selected as follows: 6 Calman^®^ (Manufacturer company: Ativus Farmacêutica Ltda, five batches), 6 Pasalix^®^ (Manufacturer company: Marjan Indústria e Comércio Ltda, five batches), 6 Serenus^®^ (Manufacturer company: BiolabSanus Farmacêutica Ltda, five batches); 6 Calmasyn^®^ (Manufacturer company: Cifarma Cientifica Farmaceutica Ltda, five batches), 6 Prakalmar^®^ (Manufacturer company: Aspen Pharma Industria Farmacêutica Ltda, five batches); 6 Maracugina PI^®^ (Manufacturer company: Natulab Laboratórios S.A., five batches). Herbal medicines containing Passiflora Incarnata, Crataegus and/or Salix alba in their composition were selected. In addition, six batches of each herbal medicine product was mixed in order to obtain a representative batch of them.

### Microwave Digestion Procedure and Quantification of Trace Metals Using ICP OES

For the closed system digestion process, the Brazilian pharmacopeia suggests using a sample mass between 0.1 and 0.5 g (Agência Nacional de Vigilância Sanitária/Fundação Oswaldo Cruz, 2010). We consider the sample mass of 0.25 g of each herbal medicine. In a quantity of 0.25 g of each sample, 4.5 ml of HNO_3_ (65%, Merck - Darmstadt, Germany), 2 ml of H_2_O_2_ and 10 ml of ultrapure water in each sample were added to tubes digestion. Subsequently, the samples were taken to the microwave digestion equipment (Speedwave four - Berghof, Germany). The optimized microwave digestion program is shown in Table 1.

**TABLE 1:** Program for digestion of herbal medicines used to microwave digestion equipment.

Step	Temperature (°C)	Pressure (bar)	Ramp Time (min)	Power (W)
1	150	30	10	700
2	190	35	5	1,120
3	50	25	1	0

The trace metals present in the herbal medicine samples were quantified by the Inductively Coupled Plasma Optical Emission Spectrometry (ICP OES, iCAP 6,300, Thermo Fisher Scientific - Bremen, Germany) under manufacturer recommended conditions for RF power (1.250 W), auxiliary Gas Flow (0.45 L/min), torch mode–axial, nebulizer concentric, coolant Gas Flow (12 L/min) and nebulizer pressure (20 psi). The following emission lines with low interference and high analytical signal were selected: Co (228.616 nm); Cu (324.754 nm); Cr (283.563 nm); Fe (259.940 nm); As (189.042 nm); Ba (455.403 nm); Cd (228.802 nm) and Pb (220.353 nm).

Spike-and-recovery experiments methods were used to verify and evaluate the accuracy of ICP OES. In spike and recovery, 1.0 ppm of each element (As, Ba, Cd, Co, Cr, Cu, Fe and Pb) were added (spiked) into the natural test sample matrix. The optimized procedures provided a recovery in the range of 81–99% of the analytes (Table 2).

**TABLE 2:** Spike and recovery test.

Analytes	Recovery (%)
As	82–86
Ba	81–88
Cd	85–87
Co	81–82
Cr	90–94
Cu	89–90
Fe	98–99
Pb	83–84

The detection limit (LODs) were calculated according to Ref. (Wenzl et al., 2016), that is, considering 3 times the standard deviation of the blank signal (B) divided by the slope of the calibration curve (SC): LOD = 3.9*B/SC. The limit of quantification (LOQs) were calculated as LOQ = 3.3* LOD. The linearity of the curves (external calibration) was evaluated based on the correlation coefficient (*R* ^*2*^), whose values reached 0.999 for all analytes (Table 3). The ranges of the calibration curves ranged from 0.001 ppm to 1.0 ppm.

**TABLE 3:** Calibration equations, correlation coefficients (*R* ^*2*^), LOD and LOQ obtained by external calibration.

Analytes	Equation external calibration y = ax + b	*R* ^*2*^	LOD (μg/g)	LOQ (μg/g)
As	Y = 536.28x + 0.986	0.9999	0.00138	0.00455
Ba	Y = 8,510,58x + 2,440	0.9999	0.00010	0.00032
Cd	Y = 11,069.5x + 20.41	0.9958	0.00031	0.00101
Co	Y = 7,231.9x – 3.363	0.9998	0.00022	0.00072
Cr	y = 13,131.402x + 53.815	0.9998	0.00052	0.00173
Cu	y = 26,073.113x + 54.789	0.9999	0.00140	0.00460
Fe	y = 12,399.396 + 10.593	0.9999	0.00103	0.00340
Pb	y = 955.275x + 2.659	0.9994	0.00176	0.00582

*y = intensity; a = slop; x = concentration (mg/L); b = intercept.

### Comparative Study

The concentration of (µg/g) of the metal (loid)s contents quantified in the herbal medicaments samples were compared with level of permitted concentrations of elemental impurities for individual component option (concentration limits (µg/g) for components used in oral drug products) established by Brazilian Pharmacopoeia (BF) (Agência Nacional de Vigilância Sanitária/Fundação Oswaldo Cruz, 2010) and United States Pharmacopoeia Convention (USP) (USP, 2021). In addition, the daily intake considered in our study (µg/day) (recommended daily dosage) was compared to permitted daily exposures for elemental impurities for drug products set by USP (USP, 2021).

Results of estimated daily intake (*EDI*) of metal (loid)s in adults and children through the consumption of herbal medicaments was compared with minimal risk levels (MRLs) (Agency for Toxic Substances and Disease Registry (ATSDR), 2020). The Agency for Toxic Substances and Disease Registry (ATSDR) established minimal risk levels (MRLs) to help in the identification of substances that may be of concern at hazardous waste sites. An MRL (mg/kg/day) is defined as “an estimate of the daily human exposure to a substance that is likely to be without an appreciable risk of adverse, non-cancer effects over a specified duration of exposure”.

Here, the estimation of daily intake (mg/kg/day) of heavy metals or metalloids due to tablet intake was calculated using the following equation (Chamannejadian et al., 2013);

$$EDI=\frac{V_{DIRx}\times C_{V}}{BW}$$ (1)

where *V*_*DIRx*_ is the rate of daily average consumption of tablet for adults and children per day (g/person/day); *C*_*V*_ is the average concentration of heavy metal (loid)s in herbal medicines (mg/kg, fresh weight). Body weight (*BW*) was considered to be 70 kg for adults (World Health Organization (WHO), 1993), and 19.25 kg for children aged 0–6 years [Brazilian Institute of Geography and Statistics (IBGE), 2006].

### Average Daily Intake of Herbal Medicines

In this study, the values of *V*_*DIRx*_ were considered according to the daily intake set by the instructions for each brand of herbal medicines (See Table 4). That is, *V*_*DIRx*_ corresponds to the total rate of tablets taken per day.

**TABLE 4:** Brand of herbal medicines, mass (g) of each tablet, number of tablets and daily intake according to the package insert.

Brand	Mass of each tablet (g)	Number of tablets	*V*_*DIRx*_ -Daily intake (g/day)
Pasalix^®^	0.5404	4 tablets	2.161
Calman^®^	0.5839	8 tablets	4.671
Serenus^®^	0.3418	4 tablets	1.367
Prakalmar^®^	0.5547	2 tablets	1.109
Calmasyn^®^	0.6942	10 tablets	6.942
Maracugina^®^	0.4232	4 tablets	1.692

### Health Risk Assessment

To assess the possibility of risks due to the ingestion of herbal medicines containing heavy metals or metalloids, the calculation of chronic daily intake (*CDI*) was used. The *CDI* value (mg/kg bw/day) is obtained from the following equation (Liu et al., 2013):

$$CDI=\frac{EDI\;x\;{FE}_{r}\;x\;{DE}_{\text{total}}}{AT}$$ (2)

where *EDI* (mg/kg/day) is the estimated daily intake of metal or metalloids due to the consumption of herbal medicines (See Eq. 1); *FE*_*r*_ is the frequency of exposure (90 days/year); *DE*_*total*_ is the duration of the exposure, that is, we consider 30 years for adults (Tschinkel et al., 2020), and for children 6 years; *AT* is the average exposure time for non-carcinogenic effects in days for adults (i.e., *AT* = 30 years × 365 days/years = 10,950 days) or children (AT = 6 years × 365 days/years = 2,190 days) (Bamuwamye et al., 2015).

### Non-carcinogenic Risks

Non-carcinogenic risks due to the ingestion of heavy metals were assessed using the target hazard quotient (*THQ*) (Bamuwamye et al., 2015).

$$THQ=\frac{CDI}{RfD}$$ (3)

In Eq. 3, *CDI* is the dose of chronic daily intake of heavy metals (mg/kg/day) obtained in Eq. 2. *RfD* is the oral reference dose recommended by USEPA for the elements As (0.0003 mg/kg/day); Cd (0.003 mg/kg/day); Co (0.0003 mg/kg/day); Cr (0.003 mg/kg/day); Cu (0.04 mg/kg/day); Fe (0.70 mg/kg/day); Pb (0.0035 mg/kg/day) (US Environmental Protection Agency, 2019), and Ba (0.07 mg/kg/day) (US Environmental Protection Agency, 2004). The hazard index (HI) is the sum of the hazard quotient. The *HI* is calculated as follows:

$$\text{HI}={\text{THQ}}_{1}+{\text{THQ}}_{2}+\cdot \cdot \cdot \cdot +{\text{THQ}}_{n}$$ (4)

where 1, 2, ..., n is the total number of metal (loid)s under consideration. Regarding HI < 1, it is considered safe, however, at the level of concern when 1 < HI < 5 (Guerra et al., 2012).

### Statistical Analysis

Data on all herbal medicines were statistically represented in the form of means and standard errors of triplicates using Excel. In addition, the one-way analysis of variance (ANOVA) was used to test whether there are any statistically significant differences between the means of metal accumulation in medicinal herbs of two or more groups (Manufacture Company). The significance level was set at p < 0.05. All statistical analysis were performed using statistical package GraphPad Prism 8.0 (San Diego, California United States)

## Results

### Metal(loid)s Contents Compared With the Reference Values Given by USP and BE

Table 5 shows the values of the concentrations of metals Ba, Cd, Co, Cr, Cu, Fe, Pb and metalloid As quantified in herbal medicines based on passionflower. In addition, the results obtained in Table 5 were compared with the levels of concentration of elements impurities for individual component option established by USP (USP, 2021), and BF (Agência Nacional de Vigilância Sanitária/Fundação Oswaldo Cruz, 2010).

**TABLE 5:** Concentration of quantified chemical elements (µg/g) in herbal medicines compared with permitted concentration of elements impurities for individual component option established by USP (USP, 2021) and FB (Agência Nacional de Vigilância Sanitária/Fundação Oswaldo Cruz, 2010).

Elements	Pasalix^®^ (µg/g)	Calman^®^ (µg/g)	Serenus^®^ (µg/g)	Prakalmar^®^ (µg/g)	Calmasyn^®^ (µg/g)	Maracugina^®^ (µg/g)	Permitted concentration of elements impurities for individual component option (oral concentration-USP) (µg/g)	Permitted concentration of elements impurities (oral concentration-BF**) (µg/g)
As	1.30 ± 0.3	1.10 ± 0.4	1.30 ± 0.08	1.70 ± 0.3	1.40 ± 0.09	1.50 ± 0.008	1.5	1.5
Ba	1.20 ± 0.06	0.110 ± 0.01	< LOD	0.180 ± 0.009	0.300 ± 0.001	3.90 ± 0.11	140	—
Cd	0.010 ± 0.002	< LOD	0.050 ± 0.009	0.00700 ± 0.001	0.060 ± 0.01	0.070 ± 0.01	5*	0.5
Co	0.610 ± 0.017	0.610 ± 0.01	0.920 ± 0.06	0.560 ± 0.02	0.790 ± 0.03	0.810 ± 0.03	5	—
Cr	0.380 ± 0.02	0.210 ± 0.02	0.610 ± 0.02	0.560 ± 0.04	0.380 ± 0.008	0.680 ± 0.03	1,100	25
Cu	1.50 ± 0.08	0.710 ± 0.02	0.610 ± 0.01	4.10 ± 0.04	3.60 ± 0.06	1.50 ± 0.005	300	250
Fe	16.4 ± 0.5	8.20 ± 0.2	1,200 ± 27.9	36.8 ± 3.4	15.3 ± 1.2	123 ± 2.6	NA	400
Pb	1.40 ± 0.01	0.940 ± 0.16	1.50 ± 0.007	1.50 ± 0.02	1.50 ± 0.02	1.70 ± 0.08	0.5	1.0

< LOD - analyte concentrations were below the limits of detection. *New values for Cadmium established in Ref. (European Medicines Agency, 2019). **Brazilian Pharmacopeia, 2010 (ANVISA, 2010) (Agência Nacional de Vigilância Sanitária/Fundação Oswaldo Cruz, 2010).

NA = Not Applicable.

In Table 6, daily intake value of the analyzed herbal medicines in µg/day (calculated on the prescribed amount) are presented and compared to permitted daily exposure for elemental impurities recommended values established by United States Pharmacopeia (USP) (USP, 2021). All values taken into consideration during this study in Table 6 are presented in daily consumption according to the package insert (Table 4).

**TABLE 6:** Daily intake value (µg/day) of the analyzed herbal medicines measured here (taking recommended dosage according Table 4) compared to recommended values by United States Pharmacopeia (USP).

Elements	Daily intake for this study (µg/day) (taking recommended dosage according Table 4)						Permitted daily exposure for elemental impurities (Oral PDE) (µg/day)
	Pasalix^®^	Calman^®^	Serenus^®^	Prakalmar^®^	Calmasyn^®^	Maracugina^®^	
As	2.81	5.14	1.78	1.88	9.72	2.54	15
Ba	2.59	0.510	< LOD	0.190	2.08	6.60	1,400
Cd	0.021	< LOD	0.068	0.00900	0.43	0.12	5
Co	1.32	2.85	1.26	0.621	5.48	1.37	50
Cr	0.820	0.120	0.830	0.620	2.64	1.15	11,000
Cu	3.24	3.32	0.830	4.55	24.9	2.54	3,000
Fe	35.4	38.3	1,640	40.8	106	208	—
Pb	3.03	4.39	2.05	1.66	10.4	2.88	5

< LOD - analyte concentrations were below the limits of detection.

### EDI Values Compared With the Values of MRLs

The *EDIs* of As, Ba, Cd, Co, Cr, Cu, Fe and Pb were calculated based on the mean concentration of each metal (loid)s in each herbal medicine and the consumption rate for adults and children (Table 7). In addition, the *EDI* values for each chemical element were compared with the MRLs levels. Table 7 shows the estimated daily intake (*EDI*) of heavy metals for an individual with a body weight of 70 and 19.25 kg for adults (30 years) and children (6 years), and frequency of exposure of 90 days/year, respectively.

**TABLE 7:** Herbal medicines, estimated daily intake (*EDI*) of elements in adults and children (µg/kg/day) through the consumption of herbal medicines compared with MRLs levels.

Elements	Herbal medicines	Adults *EDI* (µg/kg/day)	Children *EDI* (µg/kg/day)	MRLs (Agency for Toxic Substances and Disease Registry (ATSDR), 2020) Oral ingestion (µg/kg/day)
As	Pasalix^®^	0.040 ± 0.009	0.15 ± 0.04	Acute: (5) Chronic: (0.3)
	Calman^®^	0.07 ± 0.03	0.27 ± 0.09	
	Serenus^®^	0.03 ± 2.0 × 10^-3^	0.09 ± 6.0 × 10^-3^	
	Prakalmar^®^	0.03 ± 4.0 × 10^-3^	0.098 ± 0.02	
	Calmasyn^®^	0.14 ± 9.77 × 10^-3^	0.5 ± 0.04	
	Maracugina*®*	0.04 ± 2.0 × 10^-4^	0.1 ± 7.0 × 10^-4^	
Ba	Pasalix^®^	0.04 ± 2.0 × 10^-3^	0.1 ± 7.0 × 10^-3^	Chronic: (200)
	Calman^®^	7.34 × 10^-3^ ± 1.0 × 10^-3^	0.03 ± 4.0 × 10^-3^	
	Serenus^®^	0	0	
	Prakalmar^®^	2.8 × 10^-3^ ± 1.0 × 10^-4^	0.01 ± 5.0 × 10^-4^	
	Calmasyn^®^	0.3 ± 2.0 × 10^-4^	0.1 ± 6.0 × 10^-4^	
	Maracugina*®*	0.09 ± 3.0 × 10^-3^	0.34 ± 0.01	
Cd	Pasalix^®^	3.1 × 10^-4^ ± 6.0 × 10^-5^	1.1 × 10^-3^ ± 2.4 × 10^-4^	Chronic: (0.1)
	Calman^®^	0	0	
	Serenus^®^	9.8 × 10^-4^ ± 2.0 × 10^-4^	3.5 × 10^-3^ ± 6.0 × 10^-4^	
	Prakalmar^®^	1.2 × 10^-4^ ± 2.0 × 10^-5^	4.4 × 10^-4^ ± 9.0 × 10^-5^	
	Calmasyn^®^	6.147 × 10^-3^ ± 1.09 × 10^-3^	0.02 ± 4.0 × 10^-3^	
	Maracugina*®*	1.8 × 10^-3^ ± 2.0 × 10^-4^	6.4 × 10^-3^ ± 9.0 × 10^-4^	
Co	Pasalix^®^	0.02 ± 5.0 × 10^-4^	0.07 ± 2.0 × 10^-3^	Intermediate: (10)
	Calman^®^	0.041 ± 7.0 × 10^-3^	0.14 ± 0.003	
	Serenus^®^	0.018 ± 1.0 × 10^-3^	0.07 ± 5.0 × 10^-3^	
	Prakalmar^®^	8.9 × 10^-3^ ± 3.0 × 10^-3^	0.03 ± 1.0 × 10^-3^	
	Calmasyn^®^	0.02 ± 6.0 × 10^-4)^	0.07 ± 2.0 × 10^-3^	
	Maracugina*®*			
Cr	Pasalix^®^	0.01 ± 6.0 × 10^-4^	0.04 ± 2.0 × 10^-3^	Chronic: (0.9) Intermediate: (5)
	Calman^®^	0.01 ± 2.0 × 10^-3^	0.05 ± 6.0 × 10^-3^	
	Serenus^®^	0.01 ± 4.0 × 10^-4^	0.04 ± 2.0 × 10^-3^	
	Prakalmar^®^	8.9 × 10^-3^ ± 6.0 × 10^-4^	0.03 ± 2.0 × 10^-3^	
	Calmasyn^®^	0.04 ± 8.0 × 10^-4^	0.14 ± 3.0 × 10^-3^	
	Maracugina*®*	0.016 ± 7 × 10^-4^	0.06 ± 3.0 × 10^-3^	
Cu	Pasalix^®^	0.05 ± 3.0 × 10^-3^	0.2 ± 9.0 × 10^-3^	Acute: (10) Intermediate: (10)
	Calman^®^	0.053 ± 2.0 × 10^-3^	0.2 ± 7.0 × 10^-3^	
	Serenus^®^	0.012 ± 2.0 × 10^-4^	0.04 ± 9.0 × 10^-4^	
	Prakalmar^®^	0.065 ± 7.0 × 10^-4^	0.2 ± 3.0 × 10^-3^	
	Calmasyn^®^	0.4 ± 7.0 × 10^-3^	1.3 ± 0.02	
	Maracugina*®*	0.04 ± 1.0 × 10^-4^	0.13 ± 4.0 × 10^-4^	
Fe	Pasalix^®^	0.51 ± 0.01	1.8 ± 0.05	ND
	Calman^®^	0.55 ± 0.013	2.0 ± 0.05	
	Serenus^®^	23.41 ± 0.5	85.0 ± 2.0	
	Prakalmar^®^	0.58 ± 0.05	2.1 ± 0.2	
	Calmasyn^®^	1.52 ± 0.12	5.52 ± 0.44	
	Maracugina *®*	2.97 ± 0.06	10.81 ± 0.23	
Pb	Pasalix^®^	0.04 ± 6.0 × 10^-4^	0.157 ± 2.0 × 10^-3^	ND
	Calman^®^	0.03 ± 5.0 × 10^-3^	7.84 × 10^-3^ ± 1.0 × 10^-3^	
	Serenus^®^	0.03 ± 1.0 × 10^-4^	0.11 ± 5.0 × 10^-4^	
	Prakalmar^®^	0.024 ± 3.0 × 10^-4^	0.09 ± 1.0 × 10^-3^	
	Calmasyn^®^	0.15 ± 2.0 × 10^-3^	0.5 ± 8.0 × 10^-3^	
	Maracugina*®*	0.04 ± 2.0 × 10^-3^	0.15 ± 7.0 × 10^-3^	

ND = Not Determined.

### Hazard Index

The hazard index (*HI*) for non-carcinogenic health effect posed by metal (loid)s in herbal medicines for adults with 70 kg body weight (30 years) and children with 19.25 kg body weight (6 years) are presented in Table 8. To evaluate the potential risk to human health through more than one heavy metal, as well as metalloid, chronic hazard index (*HI*) is obtained as the sum of all hazard quotients (*THQ*) calculated for individual heavy metals (metalloids) for a frequency of exposure of 90 days/year (See Eq. 4).

**TABLE 8:** Total hazard index (*HI*) of the elements for adults and children.

Herbal medicines	Adults	Children
	(*HI*)	(*HI*)
Pasalix^®^	0.2148 ± 0.0322	0.779 ± 0.126
Calman^®^	0.0996 ± 0.0272	0.3614 ± 0.080
Serenus^®^	0.0468 ± 2.61 × 10^-3^	0.1707 ± 9.488 × 10^-3^
Prakalmar^®^	0.1314 ± 0.0251	0.1178 ± 1.341 × 10^-3^
Calmasyn^®^	0.1958 ± 0.010	0.7093 ± 0.0399
Maracugina^®^	0.0518 ± 9.32 × 10^-4^	0.186 ± 3.304 × 10^-3^

## Discussion

### Metal(loid)s Contents Compared With Permitted Concentration of Elements Impurities

As can be seen in Table 5, the concentration of the elements in the Pasalix herbal medicine in µg/g decreases in the order of Fe > Cu > Pb > As > Ba > Co > Cr > Cd, whereas in the Calman^®^ herbal medicine was in the order of Fe > As > Pb > Cu > Co > Cr > Ba > Cd, and in the Serenus^®^ herbal medicine was Fe > Pb > As > Co > Cd > Cr = Cu > Ba. It is observed that the orders of the quantified elements for the Prakalmar^®^ herbal medicine are arranged in the following decreasing order: Fe > Cu > As > Pb > Cr = Cu > Ba > Cd. Finally, the trends of elements quantified in the Calmasyn^®^ herbal medicine was Fe > Cu > Pb > As > Co > Cr > Ba > Cd, and for the Maracugina^®^ herbal medicine Fe > Ba > Pb > Cu = As > Co > Cr > Cd. The concentration of Ba in Serenus^®^ and Cd in Calman^®^ are below the detection limit. According to ANOVA (F (5) = 0.874, *p* = 0.5065), there are no statistically significant differences between the means of the accumulations of metals in herbal medicines of different brands.

The concentrations of As in the herbal medicines Pasalix^®^ (1.30 ± 0.3 µg/g), Calman^®^ (1.10 ± 0.4 µg/g), Serenus^®^ (1.30 ± 0.08 µg/g) and Calmasyn^®^ (1.40 ± 0.09 µg/g) are close to the values established by USP and BF (1.5 µg/g) for impurities in components used in oral drug products (Table 5). On the other hand, the concentration of As in the herbal medicine Maracugina^®^ (1.50 ± 0.008 µg/g) is equal to that of USP and BF. In addition, the concentration of As in the herbal medicine Prakalmar^®^ (1.70 ± 0.3 µg/g) is above the values set by USP and BF.

The Ba concentration in the herbal medicine samples (0.110 ± 0.01–3.90 ± 0.11 µg/g) is lower than permitted concentration of impurities established by USP (140 µg/g) (Table 5). However, permitted daily exposure limits have not yet been established for Ba by BF. The lowest concentration of Ba was observed in Calman^®^, but, the highest Ba content was found in Maracugina^®^.

The concentration ranges of Cd, Co, Cr and Cu in the herbal medicine in Table 5 are lowest than those concentration of impurities set by USP (USP, 2021), and BF (Agência Nacional de Vigilância Sanitária/Fundação Oswaldo Cruz, 2010). There are no limits for Co concentrations established by BF. The results reported here for Ba, Cr, Cu and Cd are found to be lower than those concentrations found in earlier studies including herbal medicines (Lesniewicz et al., 2006).

The iron concentration of the medicinal plants ranged from 8.20 ± 0.2 µg/g to 1,200 ± 27.9 µg/g, with Calman^®^ having the lowest value and Serenus^®^ having the highest (Table 5). The Fe concentration in the Serenus^®^ was far greater than the permitted concentration of impure elements established by BF (400 µg/g). However, the permitted concentration of impure elements such as Fe has not yet been established by USP. On the other hand, the concentrations of Fe are within the ranges found in other studies for Fe (91.8 ± 6.0 µg/g - 1,032 ± 35 µg/g) (Lesniewicz et al., 2006).

The content of Pb ranged from 0.940 ± 0.16 to 1.70 ± 0.08 µg/g in the herbal medicines (Table 5). The concentration of toxic metal Pb was higher in the Maracugina^®^ and lowest in Calman^®^. The permissible limit of Pb in impurities set by USP and FB were 0.5 µg/g and 1.0 µg/g. After comparison, content of Pb in herbal medicines with those proposed by USP and BF, it is found the concentration of Pb in Calman^®^ is higher than the impurity values established by USP for Pb in impurities, but it is less than the concentration allowed by BF. On the other hand, the concentrations of Pb are within the ranges found in other studies for Pb (0.27 ± 0.09 µg/g - 2.39 ± 1.39 µg/g) (Lesniewicz et al., 2006).

### Daily Intake Value Compared to Permitted Daily Exposure for Elemental Impurities

Few studies have compared daily intake value and exposure for elemental impurities. Thus, our main interest has been to evaluate as closely as possible the exposure of Brazilian consumers to elements. According to the results given in Table 6, the daily intake of As for adults and children due to consumption of Pasalix^®^ (2.81 µg/day), Calman^®^ (5.14 µg/day), Serenus^®^ (1.78 µg/day), Prakalmar^®^ (1.88 µg/day), Calmasyn^®^ (9.72 µg/day) and Maracugina^®^ (2.54 µg/day) are below permitted daily exposure for elemental impurities set by USP, set at 15 µg/day.

Table 6 shows that the daily intake of Ba for adults and children due to consumption of Pasalix^®^ was 2.59 µ/day, Calman^®^ was 0.510 µg/day, Prakalmar^®^ was 0.190 µg/day, Calmasyn^®^ was 2.08 µg/day and Maracugina^®^ was 6.60 µg/day. The daily intake of Ba when compared to the daily exposure values allowed for elementary impurities established by USP (1,400 µg/day), are lower than those presented.

According to Table 6, the daily intake of Cd for adults and children due to consumption of Pasalix^®^, Serenus^®^, Prakalmar^®^, Calmasyn^®^ and Maracugina^®^ was 0.021 µg/day, 0.068 µg/day, 0.00900 µg/day, 0.43 µg/day and 0.12 µg/day, and it was below the permitted daily exposure for elemental impurities (5 µg/day).

The daily intake of Co for adults and children due to consumption of Calmasyn^®^ (5.48 µg/day) followed by Calman^®^ (2.85 µg/day), Maracugina^®^ (1.37 µg/day), Pasalix^®^ (1.32 µg/day), Serenus^®^ (1.26 µg/day) and Prakalmar^®^ (0.621 µg/day), are less than the permitted daily exposure for elemental impurities (50 µg/day).

The results in Table 6 also shows that daily intake of Cr for adults and children due to consumption of Calmasyn^®^ (2.64 µg/day) was far greater than in the others, followed by Maracugina^®^ (1.15 µg/day), Serenus^®^ (0.830 µg/day), Pasalix^®^ (0.820 µg/day), Prakalmar^®^ (0.620 µg/day) and the lowest value in the Calman^®^ (0.120 µg/day). In this case, all values of daily intake of Cr for human consumption are less than the permitted daily exposure for elemental impurities, set at 1,100 µg/day.

High daily intake of Cu for adults and children due to consumption was found in the Calmasyn^®^ (24.9 µg/day), followed by Prakalmar^®^ (4.55 µg/day), Calman^®^ (3.32 µg/day), Pasalix^®^ (3.24 µg/day), Maracugina^®^ (2.54 µg/day) and the lowest in the Serenus^®^ (0.830 µg/day). All values of daily intakes are below than the permitted daily exposure for elemental impurities (3,000 µg/day).

The daily intake of Fe for adults and children due to consumption of Serenus^®^ (1,640 µg/day) was much larger than the others, followed by Maracugina^®^ (208 µg/day), Calmasyn^®^ (106 µg/day), Prakalmar^®^ (40.8 µg/day), Calman^®^ (38.3 µg/day) and the lowest recorded in Pasalix^®^ (35.4 µg/day) (Table 6). There is not limit of permitted concentration of Fe impurities for individual component option set by USP. Iron toxicity from food sources is rare. In fact, in our study, we found no results on the toxicity of iron intake caused by food or the use of medicinal herbs.

According to Table 6, the daily intake of Pb for adults and children due to consumption of Calmasyn^®^ (10.4 µg/day) was greater than the others, followed by Calman^®^ (4.39 µg/day), Pasalix^®^ (3.03 µg/day), Maracugina^®^ (2.88 µg/day), Serenus^®^ (2.05 µg/day) and the lowest intake in the Prakalmar^®^ (1.66 µg/day). The daily intake of Pb for adults and children due to consumption of Calman^®^, Pasalix^®^, Maracugina^®^ and Prakalmar^®^ are below than the permitted daily exposure for elemental impurities (5 µg/day). However, the daily intake for Calmasyn^®^ is highest than the permitted daily exposure for elemental impurities.

### EDI Values Compared With the Values of MRLs

As can be seen in Table 7, the values of *EDI* of As, Cr, Co, Cd, Cu and Ba in adults and children through the consumption of Pasalix^®^, Calman^®^, Serenus^®^, Prakalmar^®^, Calmasyn^®^ and Maracugina^®^ are below the values established by the MRLs derived for acute, intermediate and chronic exposure durations.

The minimal risk levels defined by the ATSDR have not yet been established for iron. However, the National Academy of Sciences (NAS) Dietary Reference Intake (DRI) for children is 0.45 µg/g/day for ages 4–8 years and for men aged 19 years and above is 0.11 µg/g/day (RDI - Agency U.S. et al., 2006). After comparison, Estimated Daily Intake of Fe in Table 7 with those proposed by DRI, it appears that, all *EDI* of Fe in adults and children through the consumption of Pasalix^®^, Serenus^®^, Calman^®^, Prakalmar^®^, Calmasyn^®^ and Maracugina are below these values.

There are not minimal risk levels of Pb established by ATSDR (Table 7). According to the opinion of the National Institute for Public Health and the Environment (RIVM) regarding exposure to dietary lead in the Netherlands, only 0.50 µg/kg b.w. per day is sufficient for development the neurotoxicity in young children. A quantity of 1.50 and 0.63 µg/kg b.w. per day is sufficient to cause cardiovascular effects and nephrotoxicity in adults as potential critical adverse health effects of lead (Boon et al., 2016). The estimated daily intake (*EDI*) of Pb in children due to consumption of Calmasyn^®^ (0.540 ± 7.82 × 10^-3^ µg/kg/day) are greater than RIVM (Boon et al., 2016). In addition, the estimated daily intake (*EDI*) of Pb in children due to consumption of Pasalix^®^, Serenus^®^, Calman^®^, Prakalmar^®^ and Maracugina is lower than the value obtained by RIVM. On the other hand, the value of *EDI* of Pb in adults due to consumption of the herbal medicines is lower than that set by RIVM.

The values of EDI for adults and children are below of values for Pb determinate by European Food Safety Authority (EFSA), that is, average adults consumers, lead dietary exposure ranges from 0.3 to 1.24 up to 2.43 µg/kg/day, per day, and in children 0.80 to 3.10 (average consumers), up to 5.51 µg/kg/day, per day (high consumers) (EFSA Panel on Contaminants in the Food Chain (CONTAM), 2010).

### Hazard Index

In Table 8, all the hazard index (*HI*) values recorded for adults and children in this study were below 1. This means that the consumption of these herbal medicines poses no health risk due to these metals for the age group of 30 and 6 years considering the frequency of exposure of 90 days/year and the respective daily intake (Table 6).

Short- or long-term exposure to herbal medicines containing high level of heavy metals represents a health risk. Thus, the presence of elements such as As, Ba, Cd, Co, Cr, Cu, Fe and Pb in herbal medicines or medicinal plants is worrying. Contaminants found in herbal medicines are derived from plants that may have been contaminated due to exposed to water polluted with heavy metals, use of pesticides and agrochemicals and plants that grow in heavy traffics (Shaban et al., 2016; Kumar et al., 2018; Karahan et al., 2020). Other factors such as climate, temperature and humidity can contribute to the accumulation of heavy metals in the plant (Yu et al., 2013; Haidu et al., 2017).

Some herbal medicines can be toxic to humans. In Korea, combined chronic lead and arsenic poisoning was diagnosed in a patient following consumption of a Korean herbal medicine prescribed for haemorrhoids. The 33 year-old woman had malaise, severe difficulty walking, arthralgia, oedema and abdominal pain with diarrhea, in addition, analysis of the herbal medicine revealed a high lead and arsenic content (Mitchell-Heggs et al., 1990). According to clinical practice, another study described the case of lead poisoning after ingestion of Indian herbal medicine by a 37 year-old man. Laboratory analysis showed there are blood lead concentration was high, and analysis of the herbal tablets revealed a very high lead content (Dunbabin et al., 1992). We did not find studies involving Barium toxicity in humans caused by ingestion of herbal medicine.

Acute cadmium poisoning is rare. However, there is a report on cadmium toxicity resulting in acute tubulointerstitial nephritis in a 13 year-old Croatian girl who had consumed a home-made herbal remedy. According to heavy metal analysis of herbal preparations that she had taken, the level was considered insignificant according to low-average consumption (Subat-Dezulovic et al., 2002).

We did not find studies on the toxicity of cobalt due to ingestion of herbal medicines. Literature on the daily limit of Co intake that can harm humans is contradictory. However, a study with men and women who voluntarily ingested ∼1.0 mg Co/d (0.080–0.19 μg Cog^−1^ d^−1^) of a commercially available cobalt supplement over a 3 months period showed that cobalt whole blood concentrations were not associated with clinically significant changes in basic hematologic and clinical variables (Tvermoes et al., 2014). On the other hand, the United Kingdom Expert Group on Vitamins and Minerals concluded that supplementation with 1,400 μg Co/d was unlikely to cause adverse health effects in adults [EGVM (Expert Group on Vitamins and Minerals), 2003]. The European Food Safety Authority (EFSA), which helps protect consumers from food-related risks, suggested a safe intake of 600 μg Co/day for non-carcinogenic effects [European Food Safety Authority (EFSA), 2009], and a study conducted by Finley et al. (Finley et al., 2012) concluded that a chronic oral dose of 0.03 μg Co/day would be protective of non-cancer health effects in the general population for a lifetime of daily exposure to Co.

Studies of clinical cases were not found in the literature due to toxicity caused by the ingestion of herbal medicines containing a high concentration of chromium. In addition, there was a lack of data on the presence of Cr(VI) in food. All oxidative states of the chemical element chromium [Cr(VI), Cr(III), Cr(V) and Cr(IV)] are toxic to plants and humans. Trivalent chromium is a less toxic than Cr^6+^, however, the Cr (VI) is more toxic due to carcinogenic and teratogenic effects (International Agency for Research on Cancer, 1990; De Mattia et al., 2004; Shanker et al., 2005; Sun et al., 2015). Although intoxication due to ingestion of 2 or 3 g of chromium (Cr(VI)) is often legal, a 58 year-old male survived after accidental oral ingestion of 30 g/L potassium dichromate (the estimated amount of Cr ingested is about 3 g). Despite being a rare case, the patient was discharged without renal or liver failure (Goullé et al., 2012).

Unlike various chemical elements, the literature on acute and chronic exposure to copper is vast, just read the manuscript published by the National Research Council (United States) Committee on Copper in Drinking Water [National Research Council (US) Committee on Copper in Drinking Water, 2000]. However, the human data on acute poisoning are based on cases of suicidal intent or accidental consumption of copper-contaminated foods and beverages. Therefore, it is difficult to estimate the amount of copper consumed that is toxic to humans, whether in solid form, in aqueous suspension or in solution [National Research Council (US) Committee on Copper in Drinking Water, 2000]. Although some medicinal herbs have copper in their composition, case reports of intoxication due to ingestion of medicinal herbs are scarce in the literature.

Iron toxicity from accidental ingestion is a common poisoning and hazardous to children. The Annual Report of the American Association of Poison Control Centers (AAPCC) National Poison Data System reported 2.036 cases of unintentional ingestion of iron or iron salts in children 5 years of age and younger (Mowry et al., 2016; Yuen and Becker, 2021). According to studies, ingestion of 20 mg/kg to 60 mg/kg elemental iron results in moderate symptoms and above than 60 mg/kg can result in mortality (Yuen and Becker, 2021). To date, no clinical studies involving the toxicity caused by the ingestion of iron from herbal medicines have been found.

Finally, it is important to note that the quality, safety and production standards of these herbal medicines must be required before marketing. Thus, in several countries, adequate regulatory measures and quality control of herbal products for toxic heavy metals are necessary. According to WHO, without evaluation of heavy metals, the herbal medicine should not be used (WHO, 2004).

## Conclusion

The levels of the elements As, Ba, Cd, Co, Cr, Cu, Fe and Pb in herbal medicines consumed by adult and children inhabitants of Campo Grande, Brazil, were determined by ICP OES.

The concentration of As in some herbal medicine are close to values of established by regulatory agencies. In addition, the concentration of Ba, Cd, Co, Cr and Cu in herbal medicines are lower than the permitted concentration of impurities established by these regulatory agencies and found in other studies involving herbal medicines.

Unlike other elements, the level of iron in Pasalix^®^, Calman^®^, Maracugina^®^, Prakalmar^®^ and Calmasyn^®^ was lower than the permitted concentration of impurities established by the Brazilian Pharmacopoeia, but in Serenus^®^ it was higher than the Brazilian Pharmacopoeia. In addition, the concentrations of Fe are within the ranges found in other studies for Fe. The concentration of toxic metal Pb was higher in the Maracugina^®^ and lowest in Calman^®^. The concentration of Pb in Calman^®^ is higher than the impurity values established by United States Pharmacopoeia for Pb in impurities, but it is less than the concentration allowed by Brazilian Pharmacopoeia.

The daily intake of As, Ba, Cd, Co, Cr, Cu for adults and children due to consumption of Pasalix^®^, Calman^®^, Serenus^®^, Prakalmar^®^, Calmasyn^®^ and Maracugina^®^ are below permitted daily exposure for elemental impurities set by United States Pharmacopoeia. On the other hand, the daily intake of Fe for adults and children due to consumption of Serenus^®^ was much larger than the Maracugina^®^, Calmasyn^®^, Prakalmar^®^, Calman^®^ and Pasalix^®^. Daily intake of Pb for adults and children due to consumption of Calmasyn^®^ was greater than in Calman^®^, followed by Pasalix^®^, Maracugina^®^, Serenus^®^ and Prakalmar^®^. The daily intake of Pb for adults and children consumption in Calman^®^, Pasalix^®^, Maracugina^®^ and Prakalmar^®^ are below than the permitted daily exposure for elemental impurities. However, the daily intake for Calmasyn^®^ is highest than the permitted daily exposure for elemental impurities established by United States Pharmacopoeia.

The values of *EDI* of As, Cr, Co, Cd, Cu and Ba in adults and children due to consumption of Pasalix^®^, Calman^®^, Serenus^®^, Prakalmar^®^, Calmasyn^®^ and Maracugina^®^ are below the values stipulated by the MRLs.

The *EDI* of Fe in adults and children due to consumption of Pasalix^®^, Serenus^®^, Calman^®^, Prakalmar^®^, Calmasyn^®^ and Maracugina^®^ are below the values set by Dietary Reference Intake (DRI). On the other hand, the estimated daily intake (*EDI*) of Pb in children due to consumption of Calmasyn^®^ is greater than EFSA opinion. The values of *EDI* of elements in adults due to consumption of herbal medicine are lower than EFSA’s opinion.

All the hazard index (*HI*) values recorded for adults (30 years) and children (6 years) with the frequency of exposure of 90 days/year were below 1. This means a non-carcinogenic adverse effect due to this exposure pathway*.* However, the long term health risk is high and the non-carcinogenic adverse effect is not negligible. In fact, the presence of elements such as, Cd, Co, Cr and Pb can cause damage to health.

Human Health risk assessment of heavy metals and metalloids in herbal medicines used to treat anxiety should be carried out periodically by the regulatory agencies of each country. In fact, risk assessment provides resources of reduce risks.

Studies using an animal model are necessary to ascertain the possible toxicity risks due to the ingestion of herbal medicines containing heavy metals and metalloids.

## Data Availability

The raw data supporting the conclusions of this article will be made available by the authors, without undue reservation.
